# Alternative Forms of the Rey Auditory Verbal Learning Test: A Review

**DOI:** 10.1155/2004/940191

**Published:** 2005-01-27

**Authors:** Keith A. Hawkins, David Dean, Godfrey D. Pearlson

**Affiliations:** Yale University School of Medicine and the Olin Neuropsychiatric Research Center of the Institute of LivingHartford HospitalUSA

**Keywords:** Rey Auditory Verbal Learning Test, RAVLT, AVLT, alternative forms, memory, practice effects, neuropsychological testing

## Abstract

Practice effects in memory testing complicate the interpretation of score changes over repeated testings, particularly in clinical applications. Consequently, several alternative forms of the Auditory Verbal Learning Test (AVLT) have been developed. Studies of these typically indicate that the forms examined are equivalent. However, the implication that the forms in the literature are interchangeable must be tempered by several caveats. Few studies of equivalence have been undertaken; most are restricted to the comparison of single pairs of forms, and the pairings vary across studies. These limitations are exacerbated by the minimal overlapping across studies in variables reported, or in the analyses of equivalence undertaken. The data generated by these studies are nonetheless valuable, as significant practice effects result from serial use of the same form. The available data on alternative AVLT forms are summarized, and recommendations regarding form development and the determination of form equivalence are offered.

## 1. Introduction

The assessment of change is often critical within the neuropsychological evaluation, whether in research (e.g., in determining the efficacy of treatment) or clinical settings (e.g. in delineating course, or rehabilitation results). The repeated use of instruments confounds this assessment, since practice effects – gains in performance due to prior experience with the test – have been demonstrated for many measures [9]. Practice effects are especially likely with memory testing, since the learning that occurs during the initial assessment frequently carries over to later assessments [3]. Sizeable practice effects have been demonstrated even when the gap between testings extends to months or longer [12], including within patient populations where gains are unexpected (e.g., chronic schizophrenia [10]). Cumulative gains over four administrations of the same Auditory Verbal Learning Test form at yearly intervals have been demonstrated in a mixed HIV seronegative and seropositive male sample [25]. These gains occur despite the increasing probability of ceiling effects on higher scores.

Although the assessment of verbal memory is ubiquitous within neuropsychology, many commonly used tests lack alternative forms. Multiple forms have been developed for the Hopkins Verbal Learning Test-Revised [2], a test primarily developed for older populations. A task with broader utility, the California Verbal Learning Test, appears to be accompanied by no more than one alternative form in either its original or revised version [5,6].

In contrast, several alternative forms have been developed for the Rey Auditory Verbal Learning test (AVLT). The AVLT is widely used (as evidenced by 597 PSYCHINFO citations as of mid-2004), yet no single review of the forms available, or of the equivalence of these, exists in the literature. The purpose of this report is: (i) to identify AVLT alternative forms; (ii) to present equivalence data to facilitate form selection

ISSN 0953-4180/04/$17.00 © 2004 – IOS Press and the authors. All rights reserved in serial evaluations (whether in clinical or research applications); and (iii) to present recommendations regarding the generation of alternative forms based upon shortcomings evident in the AVLT literature.

## 2. Method

### 2.1. Data location

Literature searches using PSYCINFO and MEDLINE for combinations of search terms including variants on the test name (Rey Auditory Verbal Learning Test, RAVLT or AVLT), verbal learning, verbal memory, memory, practice, practice effect, and repeat test(ing) were employed. References were also obtained from compendiums of neuropsychological tests [16,23]. The reference lists of relevant articles were examined for further literature.

### 2.2. Literature review

The approaches adopted in the determination of form equivalence varied across studies. To facilitate reader understanding of these varied methodologies, synopses of the located studies are presented.

As noted by Mitrushina et al. [16], the administration of the AVLT varies somewhat. Most studies, however, reference the administration procedures described in Lezak [13], with common elements being five presentations with recall of a 15-word list (List A), followed by a single presentation of a second "interference" list with recall. A sixth recall of List A immediately follows. Trials to test delayed recall (usually after 30 minutes) and recognition (identification of List A words from a larger set) are also commonly employed.

### 2.3. Data

Articles were examined for data pertaining to form equivalence, including study design, correlation of variables across forms, test difficulty level, and item characteristics, such as word use frequency.

### 2.4. Data reformulation

Total score for the learning trials was often absent and were generated where possible by the summing of means for trials 1–5.

Meaningful comparison data or statistical findings are lacking for several variables. Correlation coefficients between total recall scores, or between list B performances, or delayed recall scores, were seldom reported.

Word usage frequencies were determined in two ways. Kucera and Francis [11] was consulted for individual word frequencies; means and standard deviations were then calculated for each form. In the second method, Thorndike and Lorge ratings were consulted to determine how many high usage words (A and AA) are present in each list. "A" rated words occur between 50 to 100 times within one million words; "AA" words occur at least 100 times per million [24].

Insufficient data are available to determine whether these frequency ratings are related to AVLT form difficulty. Since word usage and likelihood of recall have been related [1], these data may nevertheless be of interest to readers selecting lists for test-retest employment.

## 3. Results

### 3.1. Forms located

Seven AVLT forms were accompanied in the literature by sufficient information to allow for estimations of equivalence. These forms are identified by the numbers assigned in Table 1, within which basic characteristics are presented.

### 3.2. Synopses of studies

Table 2 summarizes sample data, study design, scores, and correlations among AVLT forms, by study. The following study synopses elaborate on each study.

### 3.3. Ryan et al. [21]

Ryan et al. assessed the equivalency of the original and Lezak [13] alternative forms.

Table 1

Forms and item characteristics

| Form[1] & Source | List A Items (learning trials) | Word Frequency Mean (SD)[2] | Word Frequency: Thorndike & Lorge ratings[3] | Length in letters Mean | List B Items (interference list) | Word Frequency Mean (SD)[2] | Word Frequency: Thorndike & Lorge ratings[3] | Length in letters Mean | Comments |
|---|---|---|---|---|---|---|---|---|---|
| Form 1 Rey [19] | Drum, curtain, bell, coffee, school, parent, moon, garden, hat, farmer, nose, turkey, color, house, river | 119.5 (178.5) | 9 AA 4 A | 5.1 | Desk, ranger, bird, shoe, stove, mountain, glasses, towel, cloud, boat, lamb, gum, pencil, church, fish | 55.8 (86.3) | 7 AA 2 A | 5.0 | |
| Form 2 Lezak [13] | Book, flower, train, rug, meadow, harp, salt, finger, apple, chimney, button, log, key, rattle, gold | 39.8 (50.5) | 6 AA 3 A | 4.9 | Bowl, dawn, judge, grant, insect, plane, county, pool, seed, sheep, meal, coat, bottle, peach, chair | 56.7 (42.9) | 4 AA 8 A | 4.8 | |
| Form 3 Shapiro & Harrison [22] | Street, grass, door, arm, star, wife, window, city, pupil, cabin, lake, pipe, skin, fire, clock | 122.6 (121.3) | 11 AA 4 A | 4.5 | Baby, ocean, palace, lip, bar, dress, steam, coin, rock, army, building, friend, storm, village, cell | 66.1 (45.8) | 10 AA 5 A | 4.9 | |
| Form 4 Shapiro & Harrison [22] | Tower, wheat, queen, sugar, home, boy, doctor, camp, flag, letter, corn, nail, cattle, shore, body | 113.1 (145.5) | 9 AA 6 A | 4.7 | Sky, dollar, valley, butter, hall, diamond, winter, mother, christmas, meat, forest, gold, plant, money, hotel | 91.3 (73.1) | 12 AA 3 A | 5.5 | "Gold" in list B is also in Form 2, list A |
| Form 5 Crawford et al. [3] | Doll, mirror, nail, sailor, heart, desert, face, letter, bed, machine, milk, helmet, music, horse, road | 104.5 (105.0) | 9 AA 3 A | 5.0 | Dish, jester, hill, coat, tool, forest, water, ladder, girl, foot, shield, pie, insect, ball, car | 93.9 (124.7) | 8 AA 2 A | 4.6 | "Letter" in list A is also in Form 4, list A. "Nail" in list A is also in Form 4, list A. "Coat" in list B is also in Form 2, list B. "Forest" in list B is also in Form 4, list B. "Insect" in list B is also in Form 2, list B. |
| Form 6 Majdan et al. [15] | Violin, tree, scarf, ham, suitcase, cousin, earth, stairs, dog, banana, town, radio, hunter, bucket, field | 71.4 (82.7) | 5 AA 2 A | 5.2 | Orange, table, toad, corn, bus, chin, beach, soap, hotel, donkey, spider, money, book, soldier, padlock | 68.7 (84.9) | 4 AA 4 A | 5.0 | "Book" in list B is also in Form 2, list A. "Corn" in list B is also in Form 4, list A. "Hotel" in list B is also in Form 4, list B. "Money" in list B is also in Form 4, list B. |
| Form 7 Geffen et al. [8] | Pipe, wall, alarm, sugar, student, mother, star, painting, bag, wheat, mouth, chicken, sound, door, stream | 94.6 (91.2) | 9 AA 5 A | 5.2 | Bench, officer, cage, sock, fridge, cliff, bottle, soap, sky, ship, goat, bullet, paper, chapel, crab | 42.3 (44.5) | 4 AA 2 A | 4.9 | "Door" in list A is also in Form 3, list A. "Mother" in list A is also in Form 4, list B. "Pipe" in list A is also in Form 3, list A. "Wheat" in list A is also in Form 4, list A. "Sugar" in list A is also in Form 4, list A. "Star" in list A is also in Form 3, list A. "Bottle" in list B is also in Form 2, list B. "Sky" in list B is also in Form 4, list B. "Soap" in list B is also in Form 6, list B. |

[1]From numbering is for clarity of exposition and does not necessarily correspond to those applied in original sources.
[2]Word frequencies based upon Kucera and Francis [11].
[3]Thorndike and Lorge [24]. Indicates number of "AA" (100+ occurrences per million words) and "A" (50 to 100) words in the list.

Table 2

Form equivalence data

| Forms Compared | Sample | Design | Retest delay | Recall Total Score[1] | Total Recall *r* | List B Score | List B *r* | Post-Interference List A Recall Score | Post-Interference List A Recall *r* | Delayed Recall Score | Delayed Recall *r* | Recognition Score | Recognition *r* |
|---|---|---|---|---|---|---|---|---|---|---|---|---|---|
| 1 & 2<br>Ryan et al. [21] | N = 85<br>heterogeneous patients<br>M age = 45.9<br>M education = 11.9 yrs<br>M IQ = 89.7 | Counter-balanced | M = 140 minutes | 1: 35.55<br>2: 32.81 | .77 | | | 1: 6.18 (3.75)<br>2: 5.44 (3.27) | .66 | | | 1: 9.82 (3.64)<br>2: 10.39 (2.91) | .65 |
| 1 & 2<br>Delaney et al. [4] | N = 42<br>M age = 45.8<br>M education = 12.8 yrs | All s's tested with Form 1 then Form 2 | "Approx. one month" | *Sum of trials 1, 3 & 5 only:*<br>1: 27.7<br>2: 27.9 | | | | 1: 9.90 (3.2)<br>2: 9.90 (3.3) | | 1: 9.90 (3.1)<br>2: 9.20 (3.5) | | 1: 13.6 (1.8)<br>2: 14.0 (1.2) | |
| 1, 2, 3 & 4<br>Shapiro & Harrison [22] | N = 25<br>Undergraduates<br>M age = 19 | Counter-balanced | 2 to 13 days<br>(M = 5) | 1: 54.4<br>2: 54.1<br>3: 52.8<br>4: 55.0 | | 1: 7.20 (1.89)<br>2: 6.76 (1.59)<br>3: 7.04 (1.62)<br>4: 7.52 (1.50) | | 1: 12.00 (2.61)<br>2: 11.64 (2.12)<br>3: 11.68 (2.64)<br>4. 12.24 (2.31) | | | | | |
| 1, 2, 3 & 4<br>Shapiro & Harrison [22] | N = 42<br>Patients (mixed medical; 7 with dementia).<br>M age = 66 | Counter-balanced | 2 to 13 days<br>(M = 5) | 1: 28.6<br>2: 26.0<br>3: 27.4<br>4: 26.0 | | 1: 3.35 (1.66)<br>2: 3.41 (1.66)<br>3: 3.18 (1.55)<br>4: 3.41 (1.97) | 1 & 2: .71<br>1 & 3: .68<br>1 & 4: .74 | 1: 4.06 (3.65)<br>2: 3.29 (2.87)<br>3: 3.71 (3.67)<br>4: 4.17 (2.96) | 1 & 2: .90<br>1 & 3: .85<br>1 & 4: .88 | | | | |
| 1 & 5<br>Crawford et al. [3] | N = 60, made up of 2 groups matched for sex, age, education, NART IQ (106 & 108). | Half s's retested same form; half retested with alternate. | 27 days (± 3 days) | 1: 57.03<br>5: 55.20 | | 1: 6.90 (2.22)<br>5: 6.43 (1.89) | | 1: 11.90 (2.55)<br>2: 11.43 (2.00) | | | | 1: 25.37 (2.68)<br>5: 25.13 (2.96) | |
| 1 & 7<br>Geffen et al. [8] | N = 51<br>M age = 31.3;<br>M education = 12.2 yrs; 25 m, 26 f, NART IQ M = 115.6 (6.3). | Counter-balanced. | 6 to 14 days | 1: 51.12 (7.42)<br>7: 50.02 (7.68) | .78 | 1: 6.02 (1.73)<br>7: 5.68 (1.68) | .29 | 1: 10.88 (2.91)<br>7: 10.65 (2.71) | .70 | 1: 10.82 (2.99)<br>4: 10.33 (2.83) | .67 | 1: 13.71 (1.60)<br>7: 13.65 (1.48) | .37 |
| 1, 5 & 6<br>Majdan et al. [15] | N = 114 (100 undergraduates; 14 graduate students and hospital staff). Age 17–35, Education 12-21 years. | Each of three groups matched on gender, age, field of study, & education were tested with one form. | | 1: 61.36<br>5: 58.86<br>6: 60.85 | | 1: 8.21 (1.79)<br>5: 7.92 (1.73)<br>6: 8.63 (1.65) | | 1: 13.63 (1.78)<br>5: 12.92 (1.94)<br>6: 13.47 (1.45) | | 1: 13.68 (1.86)<br>5: 12.81 (2.05)<br>6: 13.61 (1.17) | | | |

[1] Shapiro and Harison [22], Crawford et al. [3], & Majdan et al. [15] recall total score is the sum of means for trials 1–5. $r$ =correlation coefficient between the forms.

### 3.4. Study design

A test-retest, counterbalanced design was applied with a heterogenous group of Veterans Administration Medical Center patients. Mean test-retest interval was very brief (a mean of 140 minutes).

### 3.5. Findings

Alternative form reliability coefficients were highly significant, and mean score differences of less than 1 point were found for the learning trials, post-interference trial, and recognition trials across the forms. Total recall score (the sum of trials one through five) differed by less than 3 points across the forms. When the alternative form was administered second it appeared to be slightly more difficult, though in the reverse order the two forms appear equivalent. Ryan et al. concluded that forms were equivalent measures; differences between them were considered to lack clinical significance.

### 3.6. Delaney et al. [4]

Delaney and colleagues tested 42 normal subjects (M age = 45.8, M education = 12.8) on the alternative form of the AVLT presented by Lezak [13] after initially testing them with the original form.

### 3.7. Form content

The forms consisted of the original Rey AVLT and the Lezak [13] alternative.

### 3.8. Study design

All subjects were tested on the original form and then, approximately one month later, on the Lezak form in a fixed sequence (non-counterbalanced) design.

### 3.9. Findings

Mean scores were similar across the two versions (trials 1, 3, and 5; recall of list A post the interference trial; delayed recall of list A; recognition of list A). Total recall scores (the sum of trials 1–5) were not reported; summing the means of trials 1, 3, and 5 for each form results in recall scores of 27.7 and 27.9.

The correlation coefficients between the individual trials of the original AVLT and the alternative form varied from 0.61 to 0.86 for the learning trials. Coefficients of “0.51 to 0.72 for the recall trials” were reported, but the specifics are lacking (i.e., coefficients are not identified for each of the delayed recall trials). No correlation coefficients are reported for total recall score.

### 3.10. Shapiro and Harrison [22]

Shapiro and Harrison developed two forms of the AVLT and tested these and the Lezak [13] alternative form against the original RAVLT [19] in two samples, a mixed medical/dementia group and a sample of college undergraduates.

### 3.11. Form Content

Shapiro and Harrison state that they evaluated words included in the original Rey list and Lezak alternative for occurrence in common usage, imagery, and number of syllables, and that new lists were developed using these criteria. Data to support these claims are not provided. However, per the word usage tables of Thorndike-Lorge [24], A (50–100 per million) or AA (more than 100 occurrences per million) words were used in forming their new lists. The authors state that they controlled for “obvious semantic or phonetic similarities or associations between words in the same list”.

### 3.12. Study design

All subjects were administered all four forms by the same examiner, with order counter-balanced across subjects. Inter-test intervals varied from 2 to 13 days (M = 5 days, SD = 3.6).

### 3.13. Findings

The four forms yielded individual trial scores (learning trials, interference trial, and recall of the initial list post the interference trial) that fell within 1 point. Total recall (the sum of trials 1–5), delayed recall, and recognition data are not reported. Summing the means of the learning trials for each form results in total scores that range from 52.8 to 55 for the college students, and 26 to 28.6 for the patient group (Table 2).

The correlation coefficients between the individual trials of the original AVLT and the applicable scores on the three alternative forms varied from 0.67 to 0.90 (M = 0.80). No correlation coefficients are reported for total recall score.

A general practice effect was found in the college students (i.e., they performed better on the later testing occasions despite the use of different forms). Note that retesting occurred within a mean of 5 days, and the magnitude of the effect is not reported. It was not found in the patient group. Shapiro and Harrison concluded that all four forms yielded comparable mean recall scores, and that their use "may eliminate direct practice effects", i.e., gains resulting from re-testing with the same form.

### 3.14. *Crawford et al. [3]*

Crawford et al. tested 30 normal subjects with the original Rey form, and 30 demographically matched subjects on a newly developed alternative form. Half of all subjects were retested around 4 weeks later on the form that they had initially been tested with. The remaining subjects were retested with the alternative to that used initially.

### 3.15. *Form content*

The new form consists of concrete words matched to words in the original for frequency of use per Thorndike and Lorge [24], word length, and serial position. Interference and recognition lists were similarly formed. A recognition list was generated by substitution of original Form 1 words by these new lists, and insertion of semantically or phonemically related words per Form 1 placements.

### 3.16. *Study design*

Subjects were matched for sex, age (+ 3 yr), and education (+ 1 yr) to form two groups. The groups did not differ in estimated mean IQ (106 and 108). One group was administered the original form, and the other, the new form. Subjects were retested 27 days (+ 3 days), with half of each group receiving the same form. The remaining half received the alternative version.

### 3.17. *Findings*

The group comparisons for performance on the alternative forms at the initial testing indicated no significant differences for any of the individual trials (learning trials, interference, post interference, and recognition), allowing Crawford et al. to conclude that the new version of the AVLT could be considered equivalent to the original. Total recall scores (sum of trials 1–5) were not provided. However, the sum of the means for these trials raise the possibility of a small difference in level of difficulty. The difference (original form = 57.03, new form = 55.2) of 1.83 approximates one quarter of a standard deviation of commonly reported total recall scores. Differences of this size, though small, are not necessarily trivial.

Retesting with the same form resulted in large gains, e.g., the sum of the means for trials 1–5 increased by over 7 points. Subjects retested on an alternative form, however, showed no statistically significant gains on any reported variable. The sum of means for trials 1–5 on retesting in this group is around half a point higher. Crawford et al. conclude that although large practice effects are to be expected with same form retesting, "metamemoric factors" (gains due to experience with the test format, such as improved test strategy, rather than item content ) are not operative over time spans of the order studied.

### 3.18. *Geffen et al. [8]*

Geffen et al. developed a new form and tested it against the original in 51 normal subjects.

### 3.19. *Form content*

Items within the new form were matched to the original on word frequency based upon data in Kucera & Francis [11]. The lists were also matched in number of syllables, and in semantic association properties. Interference and recognition trial lists were similarly constructed. Geffen et al. state that the new and original lists did not statistically differ on any of the word item matching variables. Frequency and word length data are reported, but semantic association data are not.

### 3.20. *Study design*

Subjects were tested on the alternative form to that administered initially from 6 to 14 days following the initial testing in a counterbalanced design. Twenty-seven subjects were tested with the original form first, and the new form later; 24 received the new form first and the original later.

### 3.21. *Findings*

No statistically significant differences were found between the forms. The 0.78 correlation for total recall score between Forms 1 and 7 is comparable to reliability coefficients reported for the same variable for single forms.

### 3.22. Majdan et al. [15]

Majdan et al. developed a new form and compared it against the original and Crawford et al. [3] forms.

### 3.23. Form content

Majdan et al. drew upon word lists generated by Rey [18] that were not used in the standard Rey list (referenced as Form 1 in this report) in forming a new form. The lists were matched on word frequency.

### 3.24. Study design

Equivalence was evaluated by comparisons of the performance of matched groups tested on separate forms: members of each group were tested just once, on one form only.

### 3.25. Findings

No statistically significant differences were found between forms.

### 3.26. Other studies

Maj et al. [14] report findings for an "Auditory Verbal Learning Test" developed for World Health Organization cross-cultural studies. The development of the test differs from the standard RAVLT approach in that words belonging to specific categories were selected to foster organized recall. This departure is of sufficient magnitude to preclude these tests from consideration within this review.

## 4. Discussion

Studies of the equivalence of AVLT alternative forms typically find that the forms studied do not differ in difficulty level. This finding, however, is tempered by several caveats. Only six studies were located, and four of these were limited to comparisons of the original Rey against one other form. Most featured a different contrasted form. The remaining studies compared the Rey original with several other forms, but, again, there is little overlap in the contrasted forms. In short, whereas the original Rey has been compared with each of six alternative forms on at least one occasion, few direct comparisons between the remaining forms exist.

The relative ranking of forms on difficulty level is further complicated by the limited over-lap that exists across studies in terms of scores reported, and in statistical findings. The data reported are typically sparse, limiting the comparisons that could otherwise be made. The predominance of trial by trial score comparisons – rather than total recall score over the five learning trials – further limits the confidence that can be placed in the literature. Individual trial scores are inherently range restricted, and are less reliable than the sum of trials. Comparisons at the trial level are considerably less likely to show statistically significant differences than comparisons of total score.

For most form comparisons no more than one study can be located. The exceptions are Forms 1 and 2 (Rey [19] and Lezak [13], respectively) and Forms 1 and 5 (Rey [19] and Crawford et al. [3]). Based upon Shapiro and Harrison [22], and Delaney et al. [4], the difficulty level of Forms 1 and 2, as reflected in the main learning trials, are similar, though the data of Ryan et al. [21], and data for a patient sample also reported by Shapiro and Harrison [22], raise the possibility that Form 1 is easier. The data presented for Forms 1 and 5 by Crawford et al. [3] and Majdan et al. [15] suggest that Form 1 may be marginally easier than 5.

Other data support the possibility that Form 1 is among the easiest of the forms. Form 1 is compared against other forms in all of the studies. In absolute terms, the learning trials total score for Form 1 is the highest score in 5 of 7 samples (Table 2). In the studies where the Form 1 total score is not highest, it is the second highest (among four forms) in one study, and virtually identical to the highest score in the other.

The differences in scores across the forms are typically small, with mean total score seldom varying by more than 2 points across forms. Large samples and multiple studies with counter-balanced designs are required to more definitively determine relative difficulty levels.

The available data are sufficient to suggest that differences between forms are sufficiently minor to be of limited concern in clinical applications, particularly if interpretation focuses upon total recall score (the most robust and arguably most meaningful score within list learning paradigms [7]). Practice effects attendant upon follow-up testing with the same form, at least over reasonably short time frames (e.g., several weeks), are likely to comfortably exceed differences in performance due to form differences. In research applications (e.g., in assessing the effect of an intervention on memory) differences in difficulty could be managed by the counterbalanced administration of alternative forms, and/or the employment of controls retested at the same intervals.

When selecting forms for use as alternatives one consideration should be whether item overlap exists. As illustrated in Table 1, numerous words appear in more than one list. Forms 5, 6, and 7 contain items in common with Form 2, an obvious confound to be considered when testing with alternatives to assess change. Other overlaps exist, with Form 7 in particular sharing many words with other lists.

Though one study [22] found a "meta-memoric", or general, practice effect (improvement on retesting with an alternative form, presumed to reflect the benefit of prior experience with the testing format), this was limited to healthy subjects who were retested within days of their initial examinations. Crawford et al. [3] did not find any such effect when retesting normal subjects four weeks after the initial exam. There are insufficient data to guide expectations for this effect, which is likely to vary across patient groups and test-retest periods. The available data suggest it is likely to be a minor consideration relative to the direct practice effects associated with retesting with the same form.

Several recommendations for form equivalence research emerge from this review. Data comparisons should not be limited to secondary variables (e.g., individual learning trials) at the expense of major variables (e.g., total score over the learning trials). The confusion created by the use of differing terms in AVLT reports could be countered by the use of operational descriptions, either within the manuscript methodology section, or the text, e.g.: "the sample obtained a mean score of 11.2 on trial 6 (recall of list A post, the interference list)."

Though the following recommendation seems so obvious as to not require stating, the existing literature proves otherwise: care must be taken to ensure that newly created lists intended to be alternative forms do not include words already employed in existing lists. Finally, differences in common usage frequency, concreteness, evocativeness (imagery), and the meaningfulness of words are sources of variance in new learning [1,17]. Creators of new word lists for memory testing should consider drawing upon data sources for these properties in drafting alternative forms, e.g. [17].
